# Gallbladder polyps and adenomyomatosis

**DOI:** 10.1259/bjr.20220115

**Published:** 2022-07-01

**Authors:** Zena C Riddell, Carmelo Corallo, Raneem Albazaz, Kieran G Foley

**Affiliations:** 1 National Imaging Academy of Wales (NIAW), Bridgend, United Kingdom; 2 Department of Radiology, Leeds Teaching Hospitals NHS Trust, St James’s University Hospital, Leeds, England; 3 Division of Cancer & Genetics, School of Medicine, Cardiff University, Wales, United Kingdom

## Abstract

Incidental findings are commonly detected during examination of the gallbladder. Differentiating benign from malignant lesions is critical because of the poor prognosis associated with gallbladder malignancy. Therefore, it is important that radiologists and sonographers are aware of common incidental gallbladder findings, which undoubtedly will continue to increase with growing medical imaging use. Ultrasound is the primary imaging modality used to examine the gallbladder and biliary tree, but contrast-enhanced ultrasound and MRI are increasingly used. This review article focuses on two common incidental findings in the gallbladder; adenomyomatosis and gallbladder polyps. The imaging features of these conditions will be reviewed and compared between radiological modalities, and the pathology, epidemiology, natural history, and management will be discussed.

## Introduction

The burden of gallbladder disease is increasing worldwide.^1^ There are more than 200,000 cases of gallbladder and biliary tract cancer globally per annum, but the number of incidental benign findings such as polyps and adenomyomatosis on imaging far exceed this each year. Although the gallbladder cancer is the most common malignancy of the biliary tree, its incidence is low with approximately 1,100 cases in the United Kingdom (UK) each year.^2^ It is important that clinicians, in particular radiologists, are aware of incidental findings of the gallbladder, especially since detection continues to increase with ongoing growth of medical imaging use. This article reviews the most common incidental findings of the gallbladder and focuses on gallbladder polyps and adenomyomatosis. We review the imaging features on different radiological modalities and the epidemiology, natural history, and management of these common incidental findings in the gallbladder.

## Methods

A detailed literature search was performed to inform this review. The MEDLINE database was searched using combinations of the terms ‘gallbladder’, ‘polyps’, and ‘adenomyomatosis’, limited to the English language and without date restriction.

### Gallbladder polyps

#### Background

Gallbladder polyps are common incidental findings on transabdominal ultrasound (TAUS) and are increasingly detected as medical imaging use increases.^3,4^ Gallbladder polyps are defined as mucosal surface elevations that protrude into the gallbladder lumen and should not be mobile or demonstrate posterior acoustic shadowing on TAUS.^5^ Gallbladder polyps can be sessile or pedunculated and categorised as either true polyps or pseudopolyps.^5–7^ True polyps most often represent adenomas of the gallbladder wall and are thought to have malignant potential.^8^ True polypoid lesions of the gallbladder can be benign and include fibromas, lipomas, and leiomyomas; or malignant, including mesenchymal neoplasms, lymphoma or metastases, but these diagnoses are far rarer than the common adenoma. Pseudopolyps have no malignant potential and commonly constitute cholesterol foci, adenomyomatosis (see section below), or inflammatory polyps. Pseudopolyps are thought to account for at least 70% of all gallbladder polyps detected by TAUS.^5^ False-positive findings also include impacted calculi adherent to the gallbladder wall, echogenic sludge, and mucosal folds. Gallbladder polyps are typically asymptomatic and hence are usually incidental findings. When symptoms are present, gallbladder polyps may be associated with intermittent right upper quadrant pain, nausea and vomiting.^9^

#### Epidemiology

Gallbladder polyps have a wide estimated prevalence in adults of 0.3–12.3%.^10^ Observational studies have demonstrated a male predilection for gallbladder polyps^9,11–13^ and a median age at diagnosis ranging from 40 to 58 years^14^.^15^ Raised body mass index (BMI) has been linked to increased prevalence of cholesterol polyps.^16^ Whilst gallbladder polyps are predominately an adult condition, they can exist in children,^17^ however, this is outside the scope of this review article. Although gallbladder polyps are prevalent, the incidence of gallbladder cancer is relatively uncommon being the 25th most common malignancy worldwide.^15^ However, gallbladder cancer has a poor prognosis with just 20% of patients diagnosed surviving for 5 years or more.^2^ Distinguishing benign findings from gallbladder cancer at an early stage is therefore critical.

#### Natural history

The adenoma–carcinoma sequence of gallbladder polyp to cancer is controversial and remains poorly understood. Studies have described the adenoma–carcinoma sequence in gallbladder polyps,^8,18^ however, a study by Wistuba et al^19^ found that adenomas did not express the molecular changes often found in dysplasia, carcinoma *in situ* and invasive malignancy, suggesting that adenomas may not be precursors to gallbladder carcinoma.

Few high-quality studies that estimate the absolute risk of malignancy in gallbladder polyps exist, but these data suggest the risk is very low. A large North American study including more than 600,000 patients with gallbladder polyps found the unadjusted gallbladder cancer rate was 11.3 per 100,000 person-years (95% confidence intervals (CI) 6.2–16.3). Rates increased with greater polyp size, from 1.3 cancers per 100,000 person-years (95% CI 0.7–6.5) in polyps less than 6 mm, to 128.2 (95% CI 9.4–217.0) in polyps 10 mm or greater.^20^ This malignancy rate is extremely low, and the authors hypothesised that, in polyps smaller than 10 mm, 95,624 ultrasound examinations after the first year would be required to detect one gallbladder cancer (equalling a rate of 1.05 per 100,000 scans). This study also demonstrated that gallbladder polyps naturally grow at a slow rate over a 20-year period, which raises concerns about the current strategy to monitor polyps using size-based criteria.

#### Risk factors for malignancy

Several clinical factors have been associated with an increased risk of malignancy in patients with gallbladder polyps and are summarised below.

#### Polyp size

There is an established association between risk of malignancy and gallbladder polyp size,^7,14,21^ but it should be noted that the risk remains relatively low even in large polyps measuring 10 mm or more.^20^ Many international guidelines use a size criterion to guide patient management.^22–24^ Most malignant polyps are larger than 10 mm. Elmasry et al found that 75% of gallbladder adenocarcinomas were 10 mm or more at diagnosis.^7^ Gallbladder polyps less than 10 mm are rarely associated with gallbladder cancer.^20^

#### Primary sclerosing cholangitis

One of the stronger associations for malignancy in patients with gallbladder polyps is the co-existence of primary sclerosing cholangitis (PSC).^25^ Several small, retrospective observational studies have shown the prevalence of gallbladder polyps to be higher than in the general population^26,27^ and with an increased risk of malignancy. Van Erp et al studied 453 patients with PSC across two centres and discovered gallbladder polyps in 16%.^26^ The gallbladder cancer rate was 8.8 (95% CI 1.8–25.7) per 1000 person-years. Said et al found malignancy present in 56% of polyps in a small study of 18 patients.^28^ Even small polyps less than 10 mm appear to be associated with an increased risk of malignancy.^27^

#### Patient age

Patient age has also been associated with the development of gallbladder cancer from polyps,^5,14^ though there are several confounding factors present when considering this association. An age criterion is also commonly used to guide management,^5,22^ but an appropriate age threshold for recommendation remains unclear. Dichotomising age at 50 years^13,29^ 60 years^14,30^ and 65 years^31,32^ have each been proposed as age-appropriate thresholds for malignant risk.

#### Number of polyps

Solitary polyps have been associated with an increased risk of malignancy in systematic review^7^ and multicentre series,^33^ suggesting that a solitary polyp is a risk factor, and should be considered in combination with other known clinical risk factors.^22^ Several other single-centre and retrospective studies have described a solitary polyp as an independent risk factor for malignancy,^21,32,34,35^ but details concerning confounding factors are often missing, thus limiting the quality of evidence.

#### Sessile morphology

A sessile polyp and eccentric gallbladder wall thickening can be difficult to differentiate on imaging. Thus, these two entities are often treated as one risk factor in guidelines.^5^ A systematic review by Bhatt et al concluded that sessile morphology of a gallbladder polyp was an independent risk factor for malignancy (odds ratio 7.32; 95% CI 4.18–12.82).^36^ Similar results were found in a study by Kwon et al.^37^ Further retrospective studies have demonstrated gallbladder wall thickening as a risk factor for malignancy.^14,38,39^ Ultimately, these studies are of low quality given the study designs and sample sizes, so the evidence for sessile morphology as a risk factor for malignancy is limited. A new GB-RADS ultrasound-based risk stratification system for gallbladder wall thickening has been proposed, however, the clinical validation and utility of this system has not been published presently.^40^

#### Ethnicity

Few studies have investigated ethnicity as a risk factor for malignancy. Aldouri et al demonstrated that Indian ethnicity was associated with increased risk of malignancy.^14^ Furthermore, Babu et al found a higher incidence of malignant gallbladder polyps in an East Asian population.^41^

#### Gallstones

Finally, the presence of gallstones as a risk factor for malignancy has been described but with limited and conflicting evidence.^4,14^ An inverse relationship between gallbladder polyps and gallstones has been described, with one study suggesting that gallstones may mechanically disrupt the formation of polyps when present.^9^ However, the evidence is not definitive and gallstones are not included as a risk factor in international guidance.^5,22^

#### Radiological investigation of gallbladder polyps

TAUS is the primary investigation for detection, diagnosis, and follow-up of gallbladder polyps and is routinely used in clinical practice worldwide. Routine use of other imaging modalities is not recommended but can be considered to aid decision making in certain circumstances with appropriate expertise.^22^

#### Imaging features

Gallbladder polyps demonstrate imaging features dependent on the radiological modality and underlying polypoidal pathology. However, imaging features overlap and this can introduce difficulty when attempting to differentiate polypoid lesions of the gallbladder.^5^ Table 1 summarises the imaging features of commonly found incidental gallbladder lesions and Table 2 compares benign and malignant imaging features.

**Table 1.** Summary of the imaging features of commonly found incidental gallbladder lesions.

Lesion	Typical age	Typical gender	Size (mm)	No of lesions	TAUS features	CT features	MRI features
Benign							
Adenoma	>60	Female	Variable (5 – 20)	Solitary	Lobulated, immobile hyperechoic lesion with no posterior acoustic shadowing Stalk usually not visualised if pedunculated Often internal vascularity at colour Doppler Homogenous enhancement at CEUS	Arterial enhancement with retention of contrast during portal venous phase	T1 hypo- to isointense, T2 hyperintense Variable signal on high b-value DWI
Adenomyomatosis	40–50	Female	<10 mm	Multiple	Mural thickening with small intramural anechoic cystic spaces which may demonstrate echogenic foci, comet tail reverberation artefact, acoustic shadowing or twinkling artefact	“Rosary sign”: intramural cystic spaces within an unenhanced hypertrophied wall with enhancing epithelium “Cotton ball sign”: fuzzy grey enhancing dots in a thickened gallbladder wall	Gallbladder wall thickening (diffuse or segmental) “Hourglass-shape” in annular adenomyomatosis Intramural lesions: - T1 hypointense -T2 hyperintense - No contrast enhancement “String of pearls sign”: T2 hyperintense foci along a thickened gallbladder wall
Gallstones	>40	Female	Variable	Variable	Mobile hyperechoic lesions with posterior acoustic shadowing Twinkling artefact on colour Doppler	Hyperattenuating (calcified gallstones) or hypoattenuating (cholesterol gallstones) to bile Most gallstones can be occult on CT as iso-attenuating to bile	T1 variable: pigmented stones are hyperintense, cholesterol stones are hypointense T2 hypointense to the bile MRCP signal voids
Tumefactive Biliary Sludge	Middle aged	No gender predilection	Variable	Variable	Intraluminal well-defined hyperechoic mass with no posterior acoustic shadowing Generally immobile Typically no internal vascularity on colour Doppler but can show twinkling artefact No enhancement with CEUS	Hyperattenuation on unenhanced phase helps differentiate from enhancing soft tissue on post-contrast phases	T1 Hyperintense Variable intensity on T2 No contrast enhancement No diffusion restriction
Cholesterol Polyps	40–50	Female	<10 mm	Multiple	“Ball on the wall sign”: immobile round/lobulated hyperechoic lesions adherent to the gallbladder wall Comet tail artefact No posterior acoustic shadow No enhancement with CEUS	No specific CT features	T2 hypointense focal lesions attached to the gallbladder wall
Inflammatory Polyps	Variable	Variable	<10 mm	Variable	Lobulated lesion of variable echogenicity No enhancement with CEUS	Non-specific features	Non-specific features
Malignant							
Gallbladder Carcinoma (Adenocarcinoma)	>60	Female	>10 mm	Solitary	Heterogenous hypoechoic lesion with irregular and ill-defined contours May present as a polyp or mural thickening Calculi and/or mural calcifications (porcelain gallbladder) can be present Heterogenous enhancement at CEUS	Hypoattenuating heterogenous lesion with areas of necrosis May be associated with lymphadenopathy, biliary/hepatic invasion, peritoneal carcinomatosis or hepatic metastases	T1 hypo- to isointense, T2 iso- to hyperintense Heterogenous enhancement Diffusion restriction with high signal on high b-value DWI
Gallbladder Metastases	>60	No gender predilection	Variable	Variable	Focal wall thickening or polypoid lesions. Possible intralesional vascularity on colour doppler flow	Similar features to the primary malignancy	Similar features to the primary malignancy

CEUS, contrast enhanced ultrasound; TAUS, transabdominal ultrasound.

**Table 2.** Summary of common benign and malignant imaging features of gallbladder lesions

	Benign features	Malignant features
TAUS / CEUS	Well-defined lobulated focal wall thickening Hyperechoic lesion which may or may not contain cystic elements Homogenous or no enhancement on CEUS	Focal wall lesion with irregular or ill-defined contours Heterogenous hypoechogenicity Heterogenous enhancement on CEUS
CT	Generally non-specific Hyperattenuating lesion on contrast CT Contrast enhancement pattern: arterial enhancement with contrast retention in portal venous phase	Generally non-specific Hypoattenuating heterogenous lesion on contrast CT
MRI	Well-defined lesion with smooth contours Unenhanced T1 and T2 are nonspecific Homogenous or no contrast enhancement Variable DWI values	Irregular morphology Unenhanced T1 and T2 are nonspecific Heterogenous contrast enhancement Diffusion restriction with high signal on high b-value DWI

CEUS, contrast-enhanced ultrasound; TAUS, trans-abdominal ultrasound.

**Figure 1.**
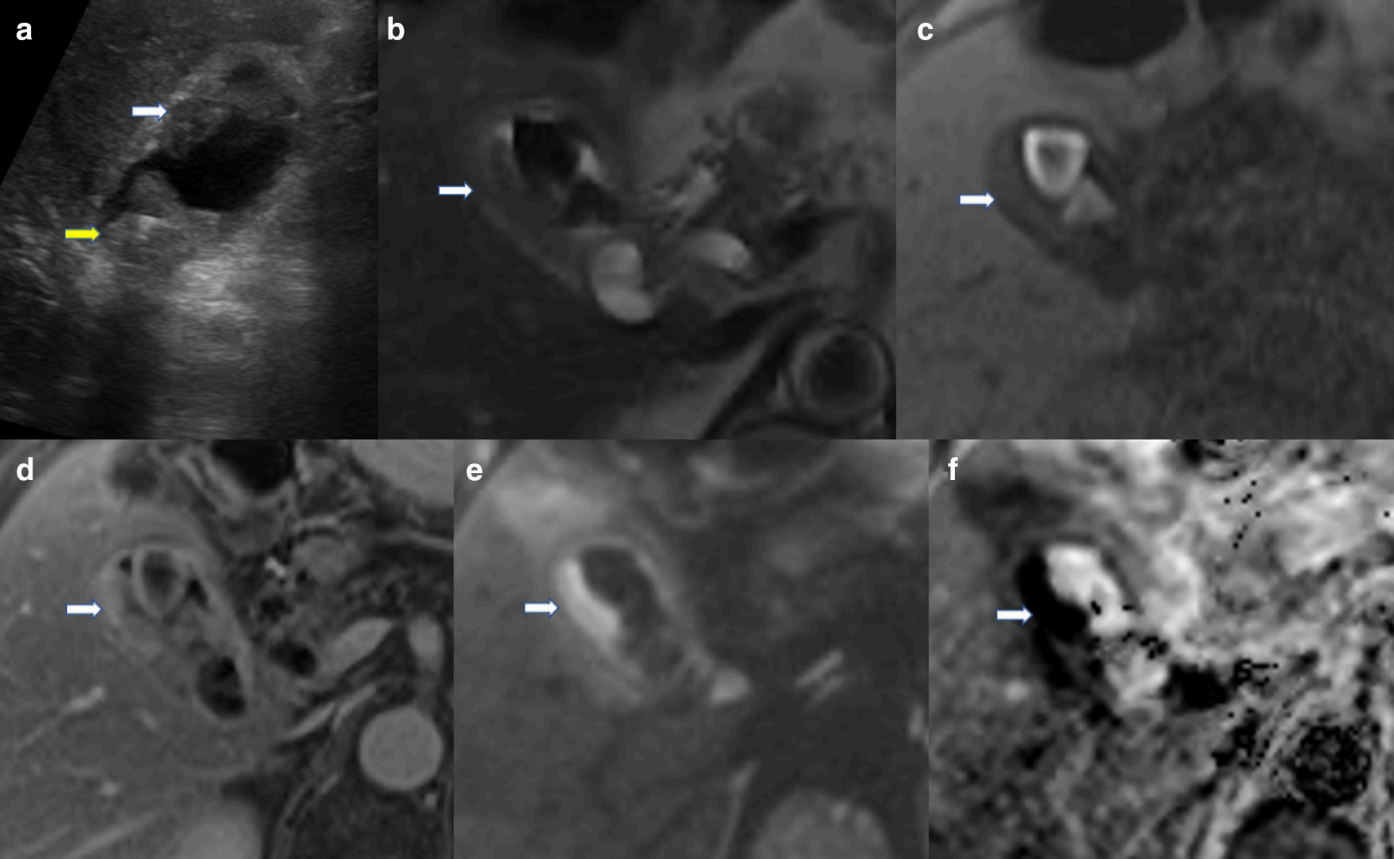
(**a**) Greyscale ultrasound of the gallbladder demonstrating multiple hyperechoic foci in the gallbladder lumen. The solid dependent areas show post-acoustic shadowing consistent with stones (yellow arrow). Solid material at the fundus is more concerning being non-dependent with irregular margin (white arrow). MRI of the same patient (**b**) T2-HASTE, (**c**) T1-VIBE unenhanced, (**d**) T1-VIBE with gadolinium, (**e**) diffusion-weighted, and (f) apparent diffusion coefficient images demonstrate low T2/high T1 signal gallstones as well as low T2/low T1 signal asymmetric soft tissue along the lateral wall of the gallbladder (white arrows). The soft tissue shows post-contrast enhancement and strong diffusion restriction consistent with malignancy. The changes in the surrounding liver were attributed to local inflammation with no focal lesion seen on image c or d. Cholecystectomy was performed and histology confirmed gallbladder adenocarcinoma with no liver involvement. HASTE, half-Fourier single-shot turbo spin-echo; VIVE, volumetric interpolated breath-hold examination.

Arguably, the most important differentiation is that between a benign or malignant gallbladder lesion. Adenocarcinoma is the most common histological cell type of gallbladder cancer^6^ and commonly presents as a large heterogeneous mass on several imaging modalities, but can present as gallbladder wall thickening, or a polypoid lesion (Figure 1). An infiltrating mass to adjacent structures is highly suggestive of malignancy. Adenocarcinoma is reported to have a nodular surface, round shape, and heterogeneous internal echoes on ultrasound.^42^ Demonstration of internal vascularity is associated with adenocarcinoma but can also be found in adenoma.^43^

Metastases to the gallbladder can occur but are uncommon.^44^ Primary malignancies include malignant melanoma, renal cell, gastric, hepatocellular, lung, and breast carcinoma. Imaging features of gallbladder metastases are difficult to differentiate from other polypoid lesions and include focal wall thickening and increased vascularity. Suspicion of metastases should be prompted by a known clinical history of malignancy.

#### Transabdominal ultrasound

**Figure 2.**
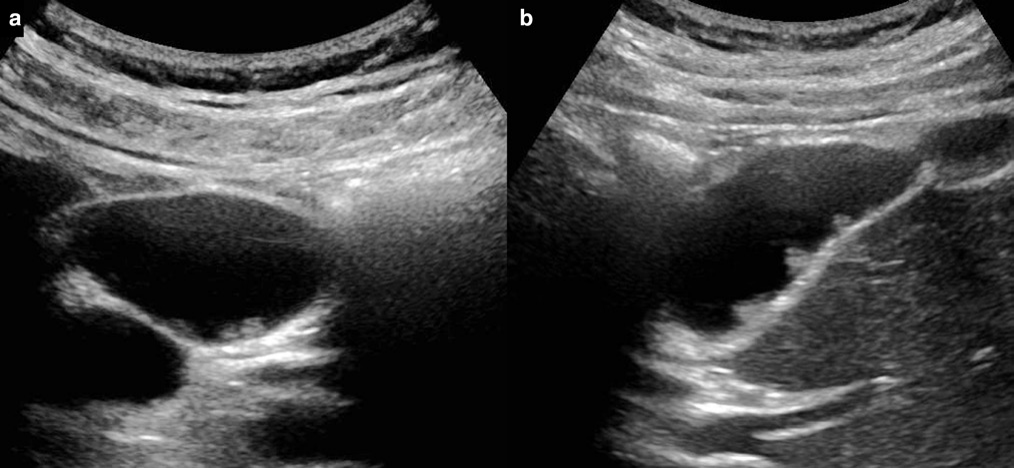
(**a, b**) Greyscale ultrasound images in transverse and longitudinal sections demonstrating multiple subcentimetre echogenic foci within the gallbladder arising from the posterior wall. Appearances are typical of small polyps (although difficult to differentiate true polyps from pseudopolyps on imaging). The polyps are non-mobile with no associated posterior acoustic shadowing helping to differentiate from stones.

Adenomas are hyperechoic compared to surrounding bile and are immobile. Adenomas can be sessile or pedunculated and lack posterior acoustic shadowing.^6^ Figure 2 If the polyp is pedunculated, the stalk is often not visualised.^45^ Adenomas vary in size and may demonstrate internal vascularity on Doppler imaging.

In contrast, pseudopolyps can vary in appearance depending on their constitution. Ultrasound features of pseudopolyps include reverberation or comet tail artefact posterior to a polypoid lesion. Cholesterol polyps may be solitary or multiple, can be pedunculated, and can have posterior acoustic shadowing.^6^ They are commonly smaller than 10 mm, may be homogeneous, or have a granular surface and an internal tiny spotted echo pattern.^42^ Cholesterol polyps may demonstrate “twinkling artefact” with the application of Doppler flow. The variation in imaging features of pseudopolyps make differentiation from true polyps challenging. Inflammatory polyps are a less common type of pseudopolyps and are associated with chronic cholecystitis.^6^ They are typically smaller, homogeneous, and either sessile or pedunculated.

TAUS is reasonable at detecting polypoid lesions of the gallbladder. A systematic review by Wennmacker et al included six studies (16,260 participants) investigating TAUS. The summary sensitivity and specificity of TAUS for the detection of gallbladder polyps was 84% (95% CI 59–95%) and 96% (95% CI 92–98%), respectively. This sensitivity and specificity support TAUS as the primary imaging modality for the diagnosis and follow-up of gallbladder polyps. However, recent studies have suggested that TAUS has a high false-positive detection rate for true polyps.^42,46–48^ One systematic review concluded that TAUS has a false-positive rate of up to 85%.^46^ Reasons for false-positive TAUS results included pseudopolyps (59%) and cholelithiasis (38%) confirmed on pathology. This low positive predictive value results in potential for unnecessary monitoring and cholecystectomy. Similarly, the diagnostic accuracy of TAUS is reduced when attempting to differentiate true polyps from pseudopolyps. The summary sensitivity was 68% (95% CI 44–85%) and the summary specificity was 79% (95% CI 57–91%). These data, coupled with the estimates that around 70% of polypoid lesions are pseudopolyps,^5^ suggest that a large proportion of gallbladder polyps may be monitored needlessly.

**Figure 3.**
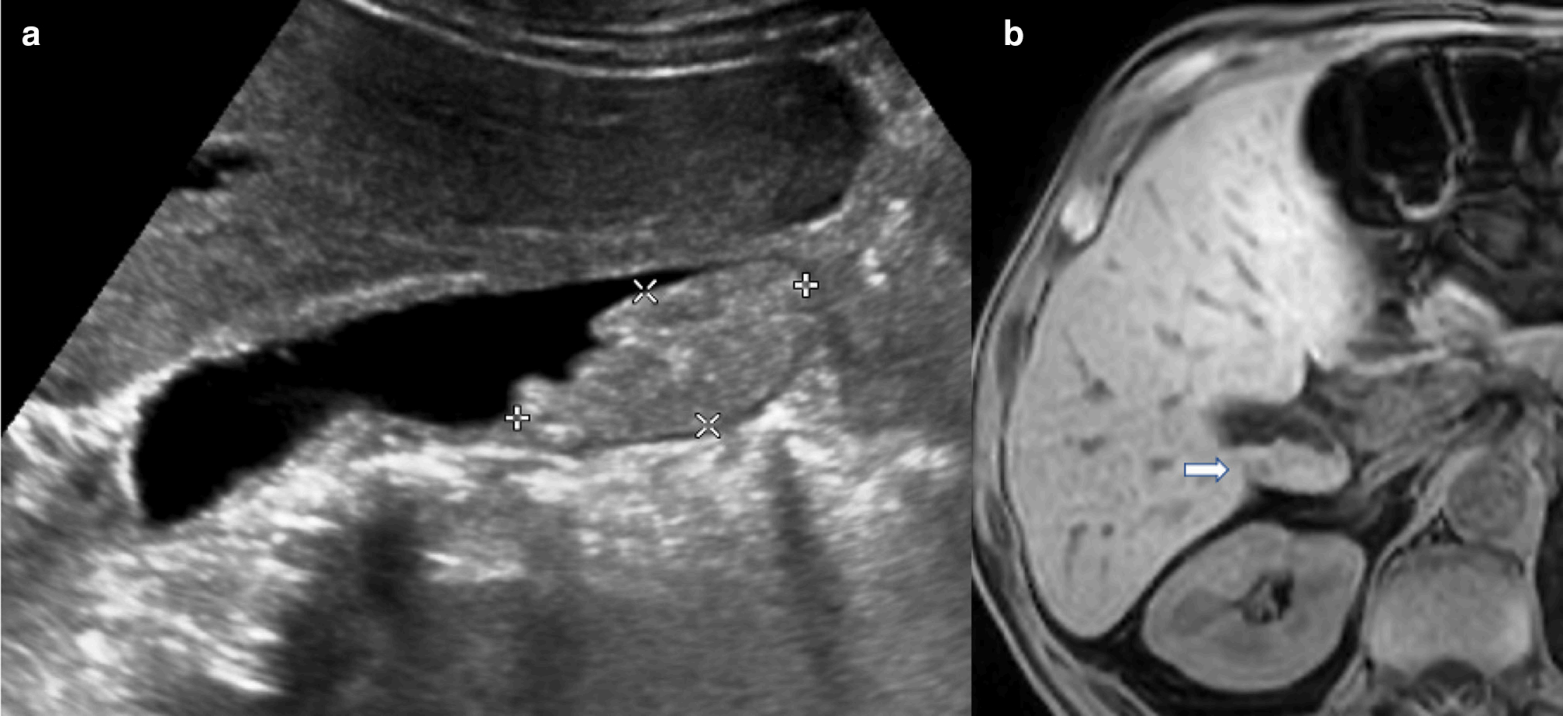
Sludge mimicking gallbladder pathology. (**a**) Greyscale ultrasound image in longitudinal section demonstrating a non-mobile gallbladder “polypoid mass”. (**b**) Unenhanced T1-VIBE image from subsequent MRI shows layering of high T1 signal within the gallbladder lumen, typical of the paramagnetic effect caused by metal ions within sludge, with no evidence of a soft tissue mass (white arrow). VIBE, volumetric interpolated breath-hold examination

**Figure 4.**
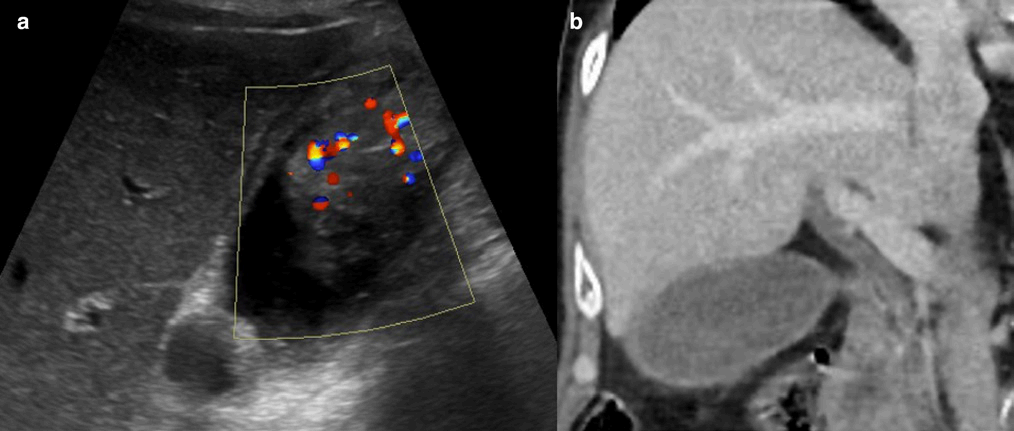
(**a**) Greyscale ultrasound images in longitudinal section demonstrating a “polypoid mass” with twinkling artefact on Doppler assessment. The twinkling artefact is thought to result from the presence of either calcifications or cholesterol deposits and it mimics high-velocity blood flow which can raise concern for a soft tissue lesion. (**b**) Subsequent coronal contrast-enhanced CT demonstrates sludge in the gallbladder lumen, appearing mildly bright with intact gallbladder wall and no evidence of a mass.

Mimics of polypoid lesions of the gallbladder include tumefactive biliary sludge and gallstones. Simple biliary sludge often demonstrates layering, can be hyperechoic, lack posterior acoustic shadowing and is mobile, but tumefactive biliary sludge can be adherent to the gallbladder wall and thus non-mobile (Figure 3). Importantly, tumefactive biliary sludge has no internal vascularity but can demonstrate twinkling artefact on Doppler assessment due to the presence of calcifications or cholesterol deposits^45^ (Figure 4). Gallstones are generally mobile, hyperechoic, and have posterior acoustic shadowing, which can be difficult to visualise in obese patients, or when the calculi are in the gallbladder neck.^3^

#### High-resolution ultrasound (HRUS)

High frequency transducers (at least 10 MHz) have better resolution, but less penetration than the low frequency transducers commonly used for TAUS.^49^ The higher resolution allows better visualisation of the gallbladder wall layers and more accurate assessment of the internal echoes of polyps than low frequency transducers but remains dependent on operator skill. HRUS is not routinely used but is being investigated as an adjunct to TAUS in the assessment of gallbladder polyps. Kim et al showed that HRUS features of neoplastic polyps included a single lobular surface, vascular core, hypoechogenicity, and internal hypoechoic foci.^49^

#### Contrast-enhanced ultrasound

**Figure 5.**
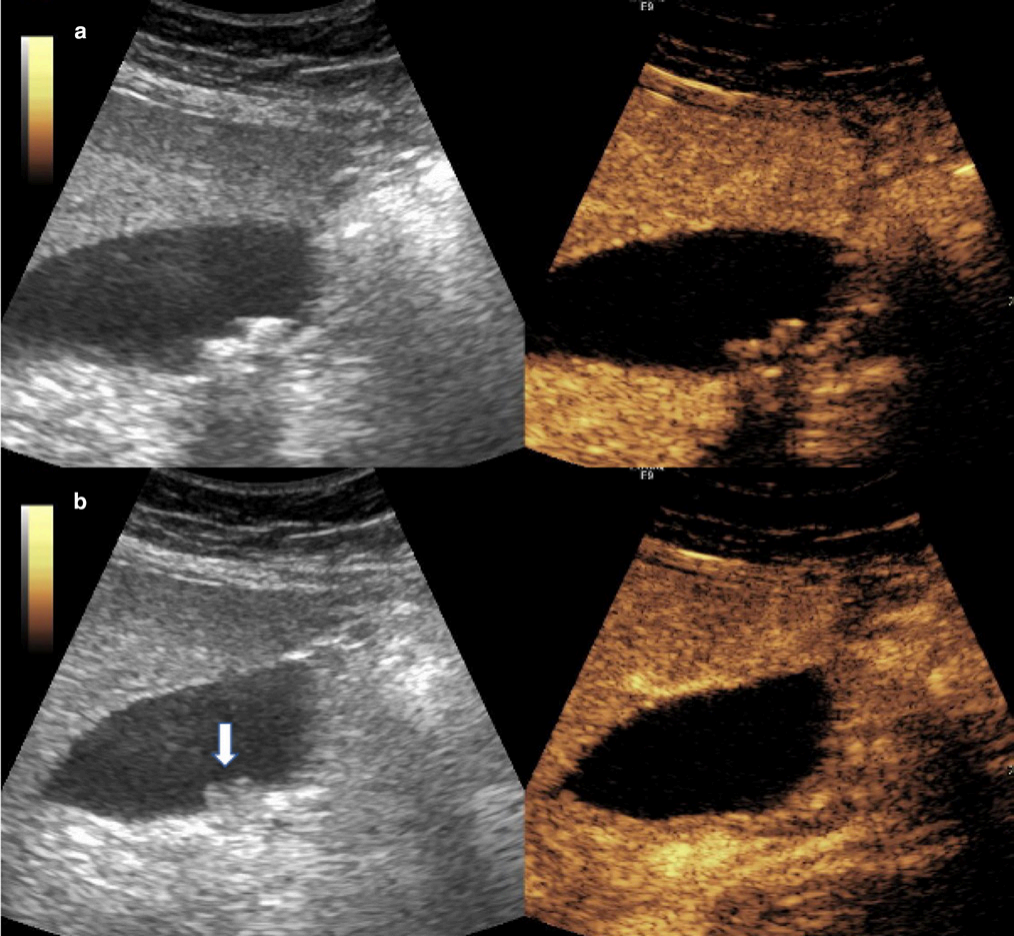
Contrast-enhanced ultrasound images for assessment of indeterminate gallbladder findings and problem solving. (**a**) Typical appearance of gallstones within the gallbladder lumen causing posterior acoustic shadowing. (**b**) A polypoid lesion at the posterior wall of the gallbladder on the greyscale image does not demonstrate enhancement on the corresponding contrast-enhanced image and is therefore in keeping with adherent sludge or pseudopolyp (white arrow).

Contrast-enhanced ultrasound (CEUS) is also available as an adjunct to TAUS. CEUS is useful in providing information on the microvascularity of a lesion, and distinguishing between vascular and non-vascular components, which can differentiate between benign and neoplastic polypoid lesions.^50^ In addition, CEUS may enable better visualisation of the polyp’s contours, morphology, size, and margins. A study by Zhang et al suggested that CEUS has a higher diagnostic accuracy than TAUS.^51^ The sensitivity of CEUS to differentiate benign and malignant polypoid gallbladder lesions was 94.1 *vs* 82.4% for TAUS, and the specificity was 95.5 *vs* 89.8%, respectively. Adenomas typically enhance homogeneously in the arterial phase and become isoechoic in the venous phase, whereas adenocarcinoma becomes hypoechoic in the venous phase. Pseudopolyps and sludge should not enhance, which can be a very useful discriminator (Figure 5)

#### Endoscopic ultrasound

Endoscopic ultrasound (EUS) is an invasive procedure that facilitates close contact with the gallbladder to produce higher resolution images. EUS enables the visualisation of the mucosa, muscularis propria, and subserosa of the gallbladder wall, and allows assessment of the morphology and surface of gallbladder polyps.^52^ Thus, EUS is theorised to be superior to transabdominal ultrasound but limited diagnostic accuracy data exist, likely to be because EUS is not routinely performed for gallbladder polyps. EUS has improved accuracy compared to TAUS for differentiating true polyps from pseudopolyps. One systematic review found summary sensitivity for EUS was 85% (95% CI 46–97%) and the summary specificity was 90% (95% CI 78–96%), compared to 68% (95% CI 44–85%) and 79% (95% CI 57–91%) for TAUS.^42^ The authors concluded that there was no significant difference in diagnostic accuracy between TAUS and EUS when differentiating between true polyps and pseudopolyps.

#### Computed tomography

In clinical practice, CT is used to diagnose, or stage, known or suspected gallbladder cancer, rather than characterise gallbladder polyps.^3^ CT is increasingly accessible, widely reported, and provides extrabiliary information.

For gallbladder polyps, a study by Kim et al demonstrated that TAUS was more sensitive than CT for the detection of gallbladder polyps.^53^ This retrospective, single-centre study showed that only 63% of polyps detected on TAUS were visible on CT. All polyps larger than 14 mm were visible on CT, whereas only 45% of polyps smaller than 13 mm were visible on CT.

Imaging features on CT may help to differentiate between neoplastic and non-neoplastic polyps.^54^ Irregular margins and sessile shape are indicative of neoplastic polyps.^55,56^ Larger polyp size (>=15 mm) is also likely to be neoplastic. Contrast enhancement on CT has also been reported to be predictive of malignancy. A retrospective study by Song et al suggested that hyperenhancement of 1–2 cm gallbladder polyps was associated with malignancy.^55^

#### Magnetic resonance imaging

**Figure 6.**
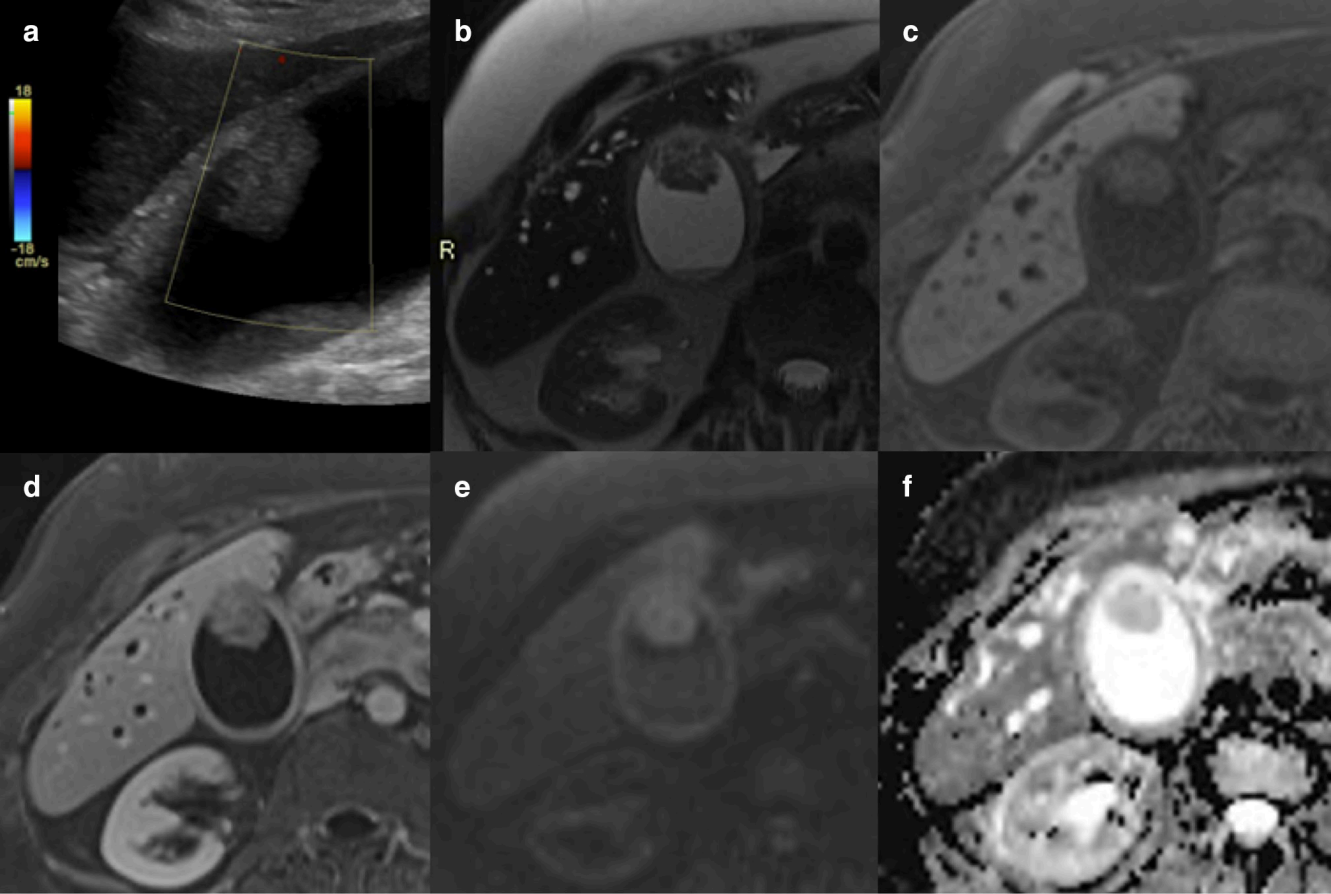
(**a**) Greyscale ultrasound demonstrating a large non-dependent polypoid hyperechoic focus within the gallbladder lumen adherent to the anterior wall. There is also echogenic debris within the gallbladder in a more dependent position along the posterior wall. Note the lack of internal vascularity, which should not be relied upon as a reassuring feature. MRI of the same lesion (**b**) T2-HASTE, (**c**) T1-VIBE unenhanced, (**d**) T1-VIBE with gadolinium, (**e**) diffusion-weighted, and (**f**) apparent diffusion coefficient images demonstrating the polypoid lesion with enhancement and diffusion restriction. No local invasion seen into the hepatic parenchyma. The gallbladder was resected and final histology showed a large adenomatous polyp with no malignant change. The patient had background PSC, which is a risk factor for gallbladder malignancy (note the dilated intrahepatic ducts). HASTE, half-Fourier single-shot turbo spin-echo; VIVE, PSC, primary sclerosing cholangitis; VIVE, volumetric interpolated breath-hold examination.

MRI is increasingly used as an imaging adjunct and problem-solving tool of the gallbladder and biliary tree.^57^ Gallbladder adenomas generally demonstrate homogeneous signal on MRI (Figure 6). A retrospective, single-centre study by Kitazume et al included 91 patients and compared diffusion-weighted imaging (DWI) with three morphological features (mass, disrupted mucosal line, and absence of two-layered pattern). When two or more morphological features were positive for malignancy the sensitivity, specificity, and accuracy were 76.9%, 84.0% and 83.0%, respectively. When morphological features were combined with apparent diffusion coefficient (ADC) values, the sensitivity, specificity and accuracy were 73.0%, 96.2% and 92.9%, respectively.^58^ Indeed, gallbladder malignancy often has higher signal on DWI compared to adenomas, whereas tumefactive sludge will not demonstrate diffusion restriction and have high T1 signal^45^ (Figure 3B).

#### Positron emission tomography (PET)

PET may also differentiate benign and malignant gallbladder wall thickening which may present incidentally on this imaging modality. A small study by Gupta et al,^59^ which included focal thickening >4 mm mimicking sessile polyps and diffuse thickening >7 mm, found that the mean standardised uptake value (SUVmax) was significantly higher in malignant (SUVmax 14.3) compared to benign thickening (SUVmax 4.5). Using a SUVmax cut-off of 5.95, the sensitivity and specificity of detecting malignancy was 92 and 79%, respectively.

#### Management of gallbladder polyps

Several international societies and organisations have developed guidelines to assist clinicians manage patients with gallbladder polyps.^5,22–24,36^ Many use clinical and size-based criteria to guide treatment options, and the frequency and duration of follow-up. Management recommendations are formed after considering the evidence base, however, a lack of prospective or randomised data significantly limit the quality of evidence concerning gallbladder polyps.

In particular, the benefit of monitoring gallbladder polyps, particularly small polyps, remains controversial and the clinical and cost-effectiveness of different management strategies are yet to be tested formally. Notably, the large North American study by Szpakowski and Tucker found that gallbladder cancer rates were similar in patients with and without polyps on initial ultrasound examination (0.053 and 0.054%, respectively).^20^

**Figure 7.**
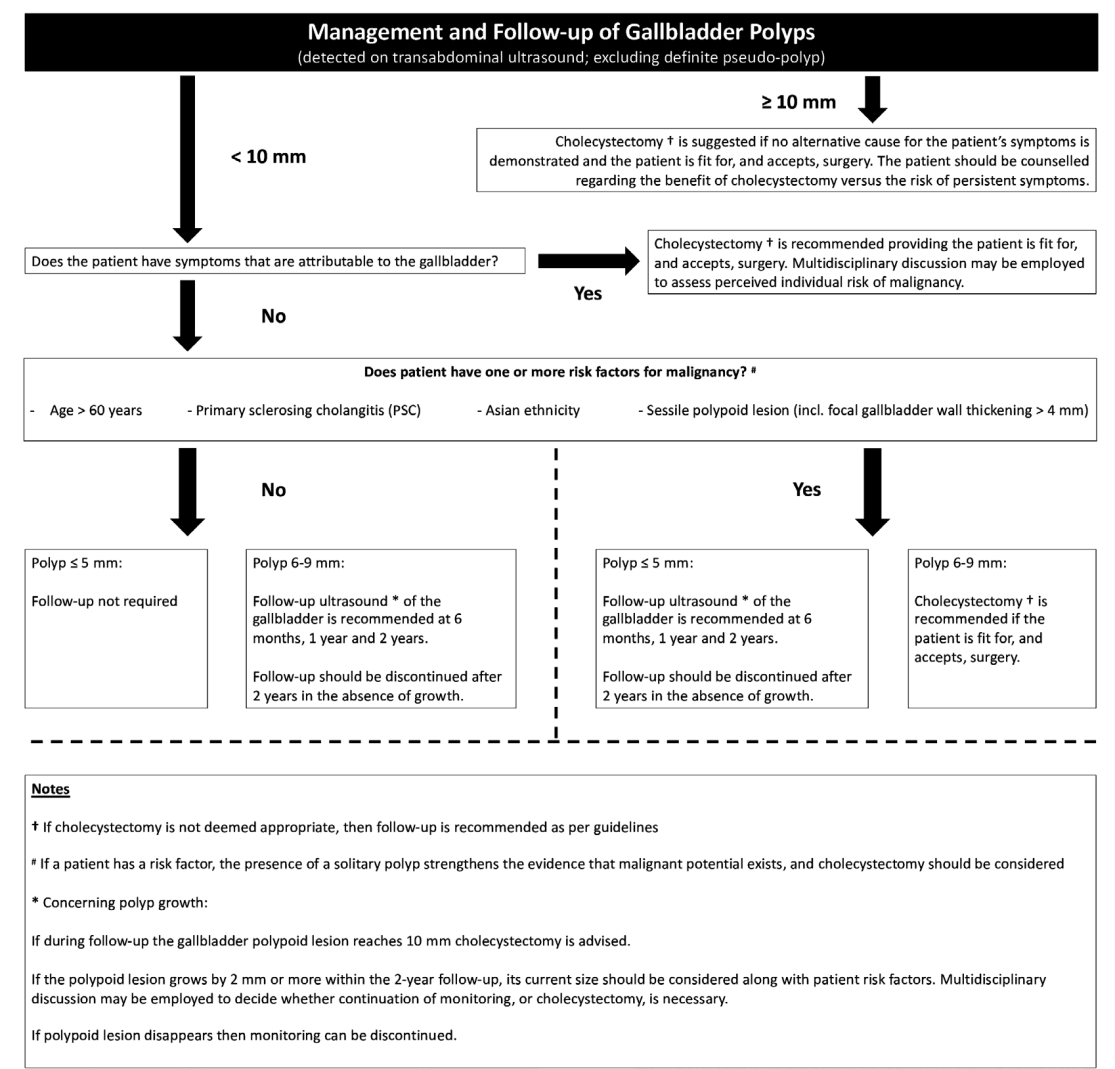
Management algorithm for gallbladder polyps detected on TAUS. Reproduced from Foley et al^22^ with permission under a Creative Commons Attribution 4.0 International License. Copyright 2021. PSC, primary sclerosing cholangitis; TAUS, transabdominal ultrasound.

Gallbladder polyp management guidance from joint European societies was updated in 2021.^5,22^ Cholecystectomy remains recommended for patients who are fit for surgery and have a polyp measuring 10 mm or more, or who have symptoms that are attributable to the gallbladder. If the polypoid lesion is less than 10 mm, then clinical risk factors should be considered. These clinical risk factors are patient age more than 60 years, PSC, Asian ethnicity, and a sessile lesion (Figure 7). Cholecystectomy is recommended for patients with clinical risk factors and a polyp measuring 6–9 mm. A solitary polyp is also concerning for malignancy and cholecystectomy should be considered alongside these risk factors. The frequency and duration of ultrasound monitoring is dependent on the size of polyp and presence of one or more risk factors. Patients without risk factors and a polyp measuring 5 mm or less no longer require follow-up.

### Adenomyomatosis

#### Background

Adenomyomatosis is a common benign, non-inflammatory hyperplastic condition that causes gallbladder wall thickening.^45,57^ Adenomyomatosis is the epithelial proliferation and hypertrophy of the gallbladder, with resulting mucosal outpouching into the thickened muscular wall.^60,61^ These mucosal outpouchings are termed Rokitansky-Aschoff sinuses^60^ and contain bile, which may dehydrate and inspissate over time, leading to precipitation of small cholesterol crystals. The crystals may result in a chronic inflammatory reaction leading to dystrophic calcification.^6^

**Figure 8.**
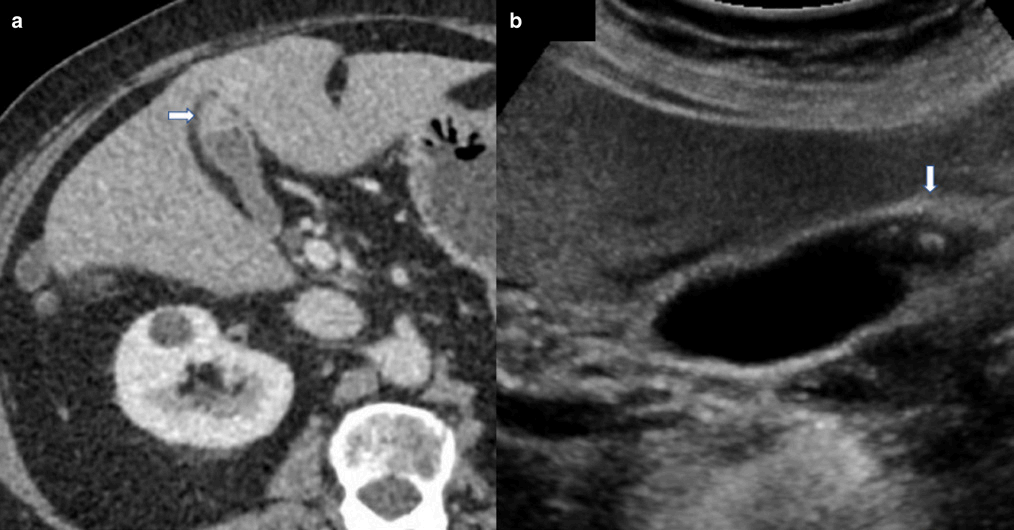
(**a**) Axial contrast-enhanced CT showing discrete focal thickening of the gallbladder fundus with normal mucosal enhancement, typical of fundal adenomyomatosis. (**b**) Greyscale ultrasound confirms nodular, hyperechoic foci studding the wall of the gallbladder causing reverberation artefact of the cholesterol crystals trapped in the mucosal sinuses—the classical “comet-tail” artefact (white arrow). Comet tail artefact can also be a feature of pseudopolyps and cholesterol polyps.

The three main morphological and radiological patterns of gallbladder wall thickening in adenomyomatosis are focal, segmental, and diffuse.^57,62^ Focal adenomyomatosis is the most common pattern and typically occurs at the fundus (Figure 8). Focal adenomyomatosis may mimic a gallbladder mass and it can be challenging to differentiate between adenomyomatosis and malignancy.^57,61^ Segmental, or annular, adenomyomatosis manifests as limited circumferential gallbladder wall thickening with luminal narrowing. Segmental adenomyomatosis usually occurs in the gallbladder body resulting in an “hourglass” appearance. Diffuse adenomyomatosis manifests as generalised gallbladder wall thickening.

#### Epidemiology

Adenomyomatosis is common in adults and its incidence is estimated to be between 2.0 and 8.7% in cholecystectomy specimens.^45^ Adenomyomatosis is generally more common in females than males. It is rare for adenomyomatosis to occur in childhood, but there have been case reports of adenomyomatosis occurring in children.^63,64^ Adenomyomatosis is usually asymptomatic but biliary colic symptoms can occur, although a confounding factor may be presence of gallstones. Adenomyomatosis is associated with gallstones in around 50% of fundal and diffuse types but can be up to 90% in the segmental type.^65^

#### Natural history

Although cases of dysplastic change and carcinoma have been reported to arise from segmental adenomyomatosis,^66^ this is rare, and thus adenomyomatosis is regarded as a benign condition. Patients with adenomyomatosis have a propensity to develop cholelithiasis and adenomyomatosis can naturally increase in size as part of its benign progression.^6^ Importantly, increasing size is not associated with malignancy.

#### Imaging features of adenomyomatosis

Adenomyomatosis demonstrates some classical features which allows the reader to make a confident diagnosis^45,60^ (Table 1). However, as is the case with gallbladder polyps, there is some overlap with malignancy that radiologists must be aware of. For instance, adenomyomatosis can manifest as a gallbladder mass, making assessment challenging.^62^ If there is clinical doubt concerning the underlying diagnosis, then early cholecystectomy should be considered.

#### TAUS

**Figure 9.**
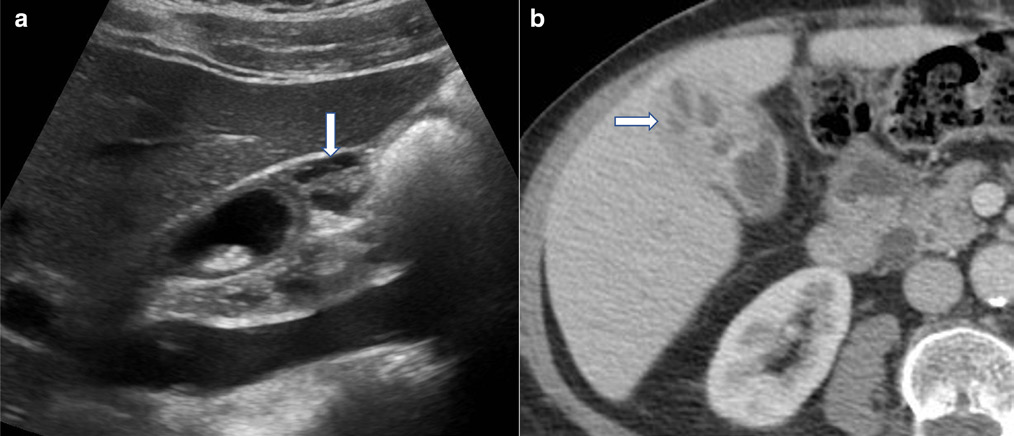
(**a**) Greyscale ultrasound showing anechoic cystic spaces in a thickened gallbladder fundus consistent with adenomyomatosis. (**b**) Axial contrast-enhanced CT also demonstrating cystic spaces within a thickened gallbladder fundus. Note the gallbladder mucosa enhances normally and appears intact. The cystic spaces are usually a sign of a benign process and are typical of adenomyomatosis.

TAUS is the primary imaging modality for the diagnosis of adenomyomatosis, but MRI offers complementary imaging features which improves diagnostic accuracy.^62^ The classic ultrasound appearance of adenomyomatosis is gallbladder wall thickening with small intramural anechoic cystic spaces^42^ (Figure 9). These intramural cystic spaces may demonstrate echogenic foci, comet tail reverberation artefact, acoustic shadowing, or twinkling artefacts due to Rokitansky-Aschoff sinuses containing biliary sludge, cholesterol crystals or calculi^45^ (Figure 7B)

#### CT

**Figure 10.**
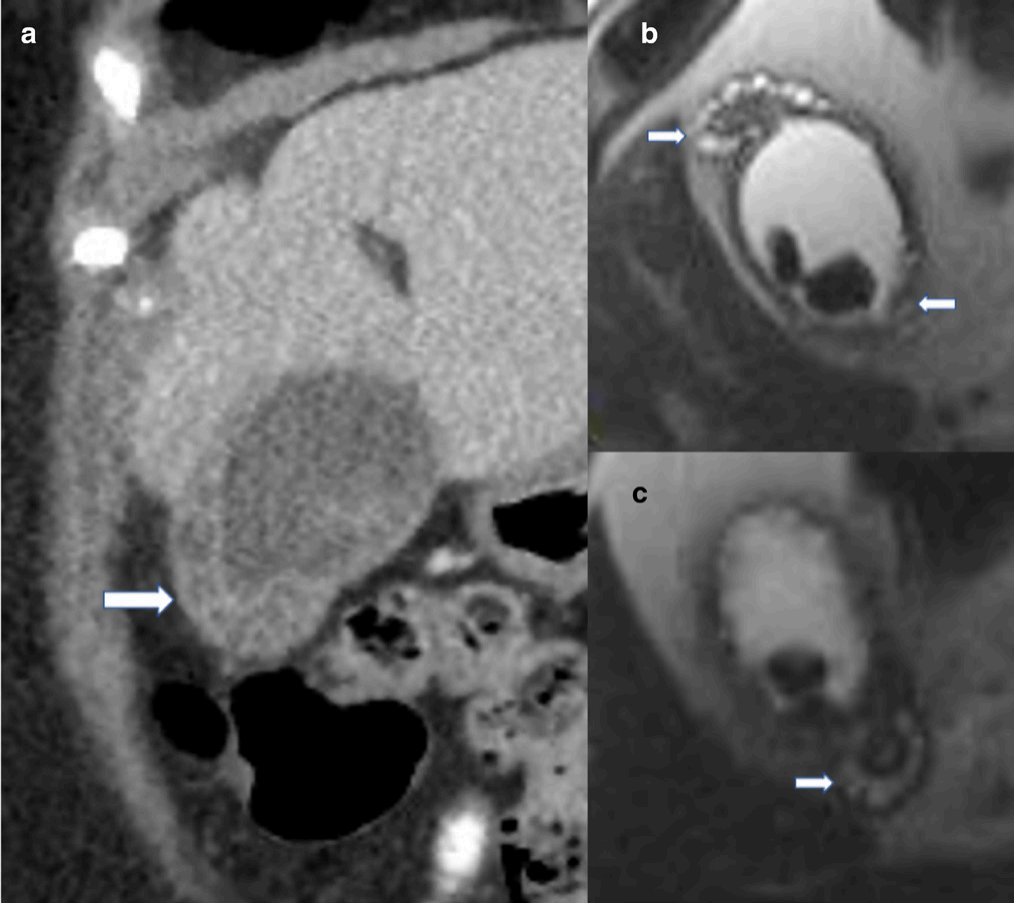
(**a**) Coronal contrast-enhanced CT, and (**b, c**) axial T2-HASTE MRI demonstrating classic “rosary bead” or “string of pearls” sign indicating the presence of Rokitansky-Aschoff sinuses within the thickened gallbladder wall seen in adenomyomatosis. Note the string of pearls appearance extends to involve the cystic duct in (c).

The “rosary sign” described on CT corresponds to the Rokitansky-Aschoff sinuses (Figure 10A). The rosary sign appears as cystic spaces within an unenhanced hypertrophied wall with an enhancing epithelium.^61,67^ Yang et al recently described the “cotton ball sign”, which may help to identify adenomyomatosis.^68^ The cotton ball sign describes fuzzy, grey enhancing dots in a thickened gallbladder wall or dotted outer border of an inner enhancing layer of the gallbladder wall on contrast-enhanced CT. However, the cotton ball sign is not seen in around 25% of cases due to limitations of CT resolution and Rokitansky-Aschoff sinuses containing biliary sludge or calculi. Overall, the differentiation of benign adenomyomatosis from malignancy can be challenging on CT, therefore if clinical suspicion of malignancy exists, then early cholecystectomy should be considered.

#### MRI

The “pearl necklace sign” on *T_2_* weighted MRI images describes the high T2 intense cystic spaces in the thickened gallbladder wall^60^ (Figure 10B and C). The pearl necklace sign may be absent when the sinuses are small (<3 mm) or contain biliary sludge or calculi. The sensitivity, specificity, and accuracy of the pearl necklace sign on MRCP is 62%, 92%, and 74%, respectively. In contrast, mucin-producing gallbladder cancer is a rare disease, but one that also demonstrates high T1 and T2 signal within small cystic spaces and has a similar appearance to adenomyomatosis. However, adenomyomatosis is much more prevalent than mucin-producing gallbladder cancer and should be treated as such.

#### CEUS

CEUS can be considered as an adjunct to TAUS if sufficient equipment and expertise are available. The typical features of adenomyomatosis on CEUS are small, non-enhancing avascular intramural foci on a background of an enhancing thickened gallbladder wall on arterial and venous phases.^69^ The serosa is intact in adenomyomatosis, whereas serosal discontinuity is concerning for malignancy.

#### HRUS

HRUS has also been investigated to differentiate between adenomyomatosis and early gallbladder cancer.^70^ A small, single-centre, retrospective study showed that features associated with adenomyomatosis included symmetrical wall thickening, intramural cystic spaces, intramural echogenic foci, and twinkling artefacts. Features including loss of multilayer pattern in the gallbladder wall and intralesional vascularity were associated with cancer.

#### EUS

EUS demonstrates similar findings to TAUS in adenomyomatosis but has higher sensitivity.^71^ However, given the invasive nature and cost of the procedure, EUS is not recommended for routine use in adenomyomatosis investigation.

#### PET

Uncomplicated adenomyomatosis does not demonstrate ^18^F-FDG PET activity. However, there may be associated chronic inflammatory change resulting in localised FDG uptake.^72^

#### Management

No national or international guidelines regarding the management of adenomyomatosis exist currently. As adenomyomatosis is benign, uncomplicated cases are generally not followed up and no specific treatment is recommended. In cases where the diagnosis is unclear, further imaging may be beneficial. Cholecystectomy may be considered in symptomatic cases, when the diagnosis is unclear, or when malignancy is suspected.

## Conclusion

Gallbladder polyps and adenomyomatosis are common incidental findings of the gallbladder. It is important that radiologists are familiar with their appearance, natural history, and management. We have reviewed the multimodality imaging features of both entities. TAUS is commonly used to diagnose and monitor these conditions but the use of MRI to image the gallbladder and biliary tree is increasing rapidly. Advances in ultrasound technology, such as HRUS and CEUS, may be used as adjuncts to TAUS in specialist centres with sufficient expertise. Whilst some imaging features are common to each pathology, there can be overlap of findings with malignancy, in which case early cholecystectomy should be considered.

## References

[1] Ouyang G, Liu Q, Wu Y, Liu Z, Lu W, Li S, et al . The global, regional, and national burden of gallbladder and biliary tract cancer and its attributable risk factors in 195 countries and territories, 1990 to 2017: A systematic analysis for the global burden of disease study 2017. Cancer 2021; 127: 2238–50. doi: 10.1002/cncr.33476 33748947

[2] Cancer Research UK . Gallbladder Cancer. 2020. Available from: https://www.cancerresearchuk.org/about-cancer/gallbladder-cancer (accessed 23 Mar 2022)

[3] Mellnick VM, Menias CO, Sandrasegaran K, Hara AK, Kielar AZ, Brunt EM, et al . Polypoid lesions of the gallbladder: disease spectrum with pathologic correlation. Radiographics 2015; 35: 387–99. doi: 10.1148/rg.352140095 25763724

[4] Park JK, Yoon YB, Kim Y-T, Ryu JK, Yoon WJ, Lee SH, et al . Management strategies for gallbladder polyps: is it possible to predict malignant gallbladder polyps? Gut Liver 2008; 2: 88–94. doi: 10.5009/gnl.2008.2.2.88 20485616 PMC2871589

[5] Wiles R, Thoeni RF, Barbu ST, Vashist YK, Rafaelsen SR, Dewhurst C, et al . Management and follow-up of gallbladder polyps: joint guidelines between the european society of gastrointestinal and abdominal radiology (ESGAR), european association for endoscopic surgery and other interventional techniques (EAES), international society of digestive surgery - european federation (EFISDS) and european society of gastrointestinal endoscopy (ESGE). Eur Radiol 2017; 27: 3856–66. doi: 10.1007/s00330-017-4742-y 28185005 PMC5544788

[6] Cocco G, Basilico R, Delli Pizzi A, Cocco N, Boccatonda A, D’Ardes D, et al . Gallbladder polyps ultrasound: what the sonographer needs to know. J Ultrasound 2021; 24: 131–42. doi: 10.1007/s40477-021-00563-1 33548050 PMC8137797

[7] Elmasry M, Lindop D, Dunne DFJ, Malik H, Poston GJ, Fenwick SW . The risk of malignancy in ultrasound detected gallbladder polyps: A systematic review. International Journal of Surgery 2016; 33: 28–35. doi: 10.1016/j.ijsu.2016.07.061 27465099

[8] Aldridge MC, Bismuth H . Gallbladder cancer: the polyp-cancer sequence. Br J Surg 1990; 77: 363–64. doi: 10.1002/bjs.1800770403 2187556

[9] Andrén-Sandberg Å . Diagnosis and management of gallbladder polyps. North Am J Med Sci 2012; 4: 203. doi: 10.4103/1947-2714.95897 PMC3359430 22655278

[10] Elmasry M, Lindop D, Dunne D, Malik H, Poston G, Fenwick S . Gallbladder polyps and the risk of malignancy: A systematic review. Br J Surg 2014; 101: 21. 10.1016/j.ijsu.2016.07.061 27465099

[11] Heitz L, Kratzer W, Gräter T, Schmidberger J . Gallbladder polyps - A follow-up study after 11 years. BMC Gastroenterol 2019; 19(1): 42. doi: 10.1186/s12876-019-0959-3 30885181 PMC6423886

[12] Jørgensen T, Jensen KH . Polyps in the gallbladder. A prevalence study. Scand J Gastroenterol 1990; 25: 281–86. 2320947

[13] Park JY, Hong SP, Kim YJ, Kim HJ, Kim HM, Cho JH, et al . Long-term follow up of gallbladder polyps. J Gastroenterol Hepatol 2009; 24: 219–22. doi: 10.1111/j.1440-1746.2008.05689.x 19054258

[14] Aldouri AQ, Malik HZ, Waytt J, Khan S, Ranganathan K, Kummaraganti S, et al . The risk of gallbladder cancer from polyps in a large multiethnic series. Eur J Surg Oncol 2009; 35: 48–51. doi: 10.1016/j.ejso.2008.01.036 18339513

[15] Sung H, Ferlay J, Siegel RL, Laversanne M, Soerjomataram I, Jemal A, et al . Global cancer statistics 2020: GLOBOCAN estimates of incidence and mortality worldwide for 36 cancers in 185 countries. CA Cancer J Clin 2021; 71: 209–49. doi: 10.3322/caac.21660 33538338

[16] Yoon JH, Kim YJ, Baik GH, Kim YS, Suk KT, Kim JB, et al . The impact of body mass index as a predictive factor of steatocholecystitis. Hepatogastroenterology 2014; 61: 902–7. 26158139

[17] Beck PL, Shaffer EA, Gall DG, Sherman PM . The natural history and significance of ultrasonographically defined polypoid lesions of the gallbladder in children. J Pediatr Surg 2007; 42: 1907–12. doi: 10.1016/j.jpedsurg.2007.07.021 18022445

[18] Kozuka S, Tsubone N, Yasui A, Hachisuka K . Relation of adenoma to carcinoma in the gallbladder. Cancer 1982; 50: 2226–34. doi: 10.1002/1097-0142(19821115)50:10<2226::aid-cncr2820501043>3.0.co;2-3 7127263

[19] Wistuba II, Miquel JF, Gazdar AF, Albores-Saavedra J . Gallbladder adenomas have molecular abnormalities different from those present in gallbladder carcinomas. Hum Pathol 1999; 30: 21–25. doi: 10.1016/s0046-8177(99)90295-2 9923922

[20] Szpakowski J-L, Tucker L-Y . Outcomes of gallbladder polyps and their association with gallbladder cancer in a 20-year cohort. JAMA Netw Open 2020; 3: e205143. doi: 10.1001/jamanetworkopen.2020.5143 32421183 PMC7235691

[21] Terzioğlu SG, Kılıç MÖ, Sapmaz A, Karaca AS . Predictive factors of neoplastic gallbladder polyps: outcomes of 278 patients. Turk J Gastroenterol 2017; 28: 202–6. doi: 10.5152/tjg.2017.16698 28316322

[22] Foley KG, Lahaye MJ, Thoeni RF, Soltes M, Dewhurst C, Barbu ST, et al . Management and follow-up of gallbladder polyps: updated joint guidelines between the ESGAR, EAES, EFISDS and ESGE. Eur Radiol 2022; 32: 3358–68. doi: 10.1007/s00330-021-08384-w 34918177 PMC9038818

[23] Bird JR, Brahm GL, Fung C, Sebastian S, Kirkpatrick IDC . Recommendations for the management of incidental hepatobiliary findings in adults: endorsement and adaptation of the 2017 and 2013 ACR incidental findings committee white papers by the canadian association of radiologists incidental findings working group. Can Assoc Radiol J 2020; 71: 437–47. doi: 10.1177/0846537120928349 32515993

[24] Sebastian S, Araujo C, Neitlich JD, Berland LL . Managing incidental findings on abdominal and pelvic CT and MRI, part 4: white paper of the ACR incidental findings committee II on gallbladder and biliary findings. J Am Coll Radiol 2013; 10: 953–56. doi: 10.1016/j.jacr.2013.05.022 24295947

[25] Buckles DC, Lindor KD, Larusso NF, Petrovic LM, Gores GJ . In primary sclerosing cholangitis, gallbladder polyps are frequently malignant. Am J Gastroenterol 2002; 97: 1138–42. doi: 10.1111/j.1572-0241.2002.05677.x 12014717

[26] van Erp LW, Cunningham M, Narasimman M, Ale Ali H, Jhaveri K, Drenth JPH, et al . Risk of gallbladder cancer in patients with primary sclerosing cholangitis and radiographically detected gallbladder polyps. Liver Int 2020; 40: 382–92. doi: 10.1111/liv.14326 31823479

[27] Torabi Sagvand B, Edwards K, Shen B . Frequency, risk factors, and outcome of gallbladder polyps in patients with primary sclerosing cholangitis: A case-control study. Hepatol Commun 2018; 2: 1440–45. doi: 10.1002/hep4.1276 30556033 PMC6287476

[28] Said K, Glaumann H, Bergquist A . Gallbladder disease in patients with primary sclerosing cholangitis. J Hepatol 2008; 48: 598–605. doi: 10.1016/j.jhep.2007.11.019 18222013

[29] Chou SC, Chen SC, Shyr YM, Wang SE . Polypoid lesions of the gallbladder: analysis of 1204 patients with long-term follow-up. Surg Endosc 2017; 31: 2776–82. doi: 10.1007/s00464-016-5286-y 28039652

[30] Shin SR, Lee JK, Lee KH, Lee KT, Rhee JC, Jang K-T, et al . Can the growth rate of a gallbladder polyp predict a neoplastic polyp? J Clin Gastroenterol 2009; 43: 865–68. doi: 10.1097/MCG.0b013e31819359aa 19398929

[31] Cha BH, Hwang J-H, Lee SH, Kim JE, Cho JY, Kim H, et al . Pre-operative factors that can predict neoplastic polypoid lesions of the gallbladder. World J Gastroenterol 2011; 17: 2216–22. doi: 10.3748/wjg.v17.i17.2216 21633532 PMC3092874

[32] Onda S, Futagawa Y, Gocho T, Shiba H, Ishida Y, Okamoto T, et al . A preoperative scoring system to predict carcinoma in patients with gallbladder polyps. Dig Surg 2020; 37: 275–81. doi: 10.1159/000503100 31722357

[33] Wennmacker SZ, van Dijk AH, Raessens JHJ, van Laarhoven C, Drenth JPH, de Reuver PR, et al . Polyp size of 1 cm is insufficient to discriminate neoplastic and non-neoplastic gallbladder polyps. Surg Endosc 2019; 33: 1564–71. doi: 10.1007/s00464-018-6444-1 30203209 PMC6484812

[34] Choi TW, Kim JH, Park SJ, Ahn SJ, Joo I, Han JK . Risk stratification of gallbladder polyps larger than 10 mm using high-resolution ultrasonography and texture analysis. Eur Radiol 2018; 28: 196–205. doi: 10.1007/s00330-017-4954-1 28687913

[35] Yang J-I, Lee JK, Ahn DG, Park JK, Lee KH, Lee KT, et al . Predictive model for neoplastic potential of gallbladder polyp. J Clin Gastroenterol 2018; 52: 273–76. doi: 10.1097/MCG.0000000000000900 28742730

[36] . Bhatt NR, Gillis A, Smoothey CO, Awan FN, Ridgway PF. Evidence based management of polyps of the gall bladder: A systematic review of the risk factors of malignancy. Surgeon. 2016;14(5):278-86. 26825588 10.1016/j.surge.2015.12.001

[37] Kwon W, Jang J-Y, Lee SE, Hwang DW, Kim S-W . Clinicopathologic features of polypoid lesions of the gallbladder and risk factors of gallbladder cancer. J Korean Med Sci 2009; 24: 481. doi: 10.3346/jkms.2009.24.3.481 19543513 PMC2698196

[38] Zhu J-Q, Han D-D, Li X-L, Kou J-T, Fan H, He Q . Predictors of incidental gallbladder cancer in elderly patients. Hepatobiliary & Pancreatic Diseases International 2015; 14: 96–100. doi: 10.1016/S1499-3872(14)60292-7 25655297

[39] Choi SY, Kim TS, Kim HJ, Park JH, Park DI, Cho YK, et al . Is it necessary to perform prophylactic cholecystectomy for asymptomatic subjects with gallbladder polyps and gallstones? Journal of Gastroenterology and Hepatology 2010; 25: 1099–1104. doi: 10.1111/j.1440-1746.2010.06288.x 20594225

[40] . Gupta P, Dutta U, Rana P, Singhal M, Gulati A, Kalra N, et al . Gallbladder reporting and data system (GB-RADS) for risk stratification of gallbladder wall thickening on ultrasonography: an international expert consensus. Abdominal Radiology. 2022;47(2):554-65. 34851429 10.1007/s00261-021-03360-w

[41] Babu BI, Dennison AR, Garcea G . Management and diagnosis of gallbladder polyps: a systematic review. Langenbecks Arch Surg 2015; 400: 455–62. doi: 10.1007/s00423-015-1302-2 25910600

[42] Wennmacker SZ, Lamberts MP, Di Martino M, Drenth JP, Gurusamy KS, van Laarhoven CJ . Transabdominal ultrasound and endoscopic ultrasound for diagnosis of gallbladder polyps. Cochrane Database Syst Rev 2018; 8. doi: 10.1002/14651858.CD012233.pub2 PMC6513652 30109701

[43] Miwa H, Numata K, Sugimori K, Kaneko T, Maeda S . Vascular evaluation using transabdominal ultrasound for gallbladder polyps. J Med Ultrason (2001) 2021; 48: 159–73. doi: 10.1007/s10396-020-01008-8 32125576

[44] Cocco G, Delli Pizzi A, Basilico R, Fabiani S, Taraschi AL, Pascucci L, et al . Imaging of gallbladder metastasis. Insights Imaging 2021; 12(1): 100. doi: 10.1186/s13244-021-01049-8 34259932 PMC8280258

[45] Yu MH, Kim YJ, Park HS, Jung SI . Benign gallbladder diseases: imaging techniques and tips for differentiating with malignant gallbladder diseases. World J Gastroenterol 2020; 26: 2967–86. doi: 10.3748/wjg.v26.i22.2967 32587442 PMC7304100

[46] Martin E, Gill R, Debru E . Diagnostic accuracy of transabdominal ultrasonography for gallbladder polyps: systematic review. Can J Surg 2018; 61: 200–207. doi: 10.1503/cjs.011617 29806818 PMC5973908

[47] Metman MJH, Olthof PB, van der Wal JBC, van Gulik TM, Roos D, Dekker JWT . Clinical relevance of gallbladder polyps; is cholecystectomy always necessary? HPB (Oxford) 2020; 22: 506–10. doi: 10.1016/j.hpb.2019.08.006 31481314

[48] Li Y, Tejirian T, Collins JC . Gallbladder polyps: real or imagined? Am Surg 2018; 84: 1670–74. 30747692

[49] Kim JH, Lee JY, Baek JH, Eun HW, Kim YJ, Han JK, et al . High-resolution sonography for distinguishing neoplastic gallbladder polyps and staging gallbladder cancer. AJR Am J Roentgenol 2015; 204: W150-9. doi: 10.2214/AJR.13.11992 25615775

[50] Bae JS, Kim SH, Kang H-J, Kim H, Ryu JK, Jang J-Y, et al . Quantitative contrast-enhanced US helps differentiating neoplastic vs non-neoplastic gallbladder polyps. Eur Radiol 2019; 29: 3772–81. doi: 10.1007/s00330-019-06123-w 30963274

[51] Zhang H-P, Bai M, Gu J-Y, He Y-Q, Qiao X-H, Du L-F . Value of contrast-enhanced ultrasound in the differential diagnosis of gallbladder lesion. World J Gastroenterol 2018; 24: 744–51. doi: 10.3748/wjg.v24.i6.744 29456413 PMC5807677

[52] Cho JH, Park JY, Kim YJ, Kim HM, Kim HJ, Hong SP, et al . Hypoechoic foci on EUS are simple and strong predictive factors for neoplastic gallbladder polyps. Gastrointest Endosc 2009; 69: 1244–50. doi: 10.1016/j.gie.2008.10.017 19249773

[53] Kim JS, Lee JK, Kim Y, Lee SM, et al . US characteristics for the prediction of neoplasm in gallbladder polyps 10 mm or larger. Eur Radiol 2016; 26: 1134–40. doi: 10.1007/s00330-015-3910-1 26188659

[54] Park KW, Kim SH, Choi SH, Lee WJ . Differentiation of nonneoplastic and neoplastic gallbladder polyps 1 cm or bigger with multi-detector row computed tomography. J Comput Assist Tomogr 2010; 34: 135–39. doi: 10.1097/RCT.0b013e3181b382d7 20118736

[55] Song ER, Chung W-S, Jang HY, Yoon M, Cha EJ . CT differentiation of 1-2-cm gallbladder polyps: benign vs malignant. Abdom Imaging 2014; 39: 334–41. doi: 10.1007/s00261-013-0071-z 24420067

[56] Furukawa H, Takayasu K, Mukai K, Inoue K, Kyokane T, Shimada K, et al . CT evaluation of small polypoid lesions of the gallbladder. Hepatogastroenterology 1995; 42: 800–810. 8847027

[57] Catalano OA, Sahani DV, Kalva SP, Cushing MS, Hahn PF, Brown JJ, et al . MR imaging of the gallbladder: A pictorial essay. Radiographics 2008; 28: 135–55. doi: 10.1148/rg.281065183 18203935

[58] Kitazume Y, Taura S-I, Nakaminato S, Noguchi O, Masaki Y, Kasahara I, et al . Diffusion-weighted magnetic resonance imaging to differentiate malignant from benign gallbladder disorders. Eur J Radiol 2016; 85: 864–73. doi: 10.1016/j.ejrad.2016.02.003 26971436

[59] Gupta V, Vishnu KS, Yadav TD, Sakaray YR, Irrinki S, Mittal BR, et al . Radio-pathological correlation of 18F-FDG PET in characterizing gallbladder wall thickening. J Gastrointest Cancer 2019; 50: 901–6. doi: 10.1007/s12029-018-0176-2 30397856

[60] Haradome H, Ichikawa T, Sou H, Yoshikawa T, Nakamura A, Araki T, et al . The pearl necklace sign: an imaging sign of adenomyomatosis of the gallbladder at MR cholangiopancreatography. Radiology 2003; 227: 80–88. doi: 10.1148/radiol.2271011378 12601186

[61] Boscak AR, Al-Hawary M, Ramsburgh SR . Adenomyomatosis of the gallbladder. RadioGraphics 2006; 26: 941–46. doi: 10.1148/rg.263055180 16702464

[62] Golse N, Lewin M, Rode A, Sebagh M, Mabrut JY . Gallbladder adenomyomatosis: diagnosis and management. J Visc Surg 2017; 154: 345–53. doi: 10.1016/j.jviscsurg.2017.06.004 28844704

[63] Agrusti A, Gregori M, Salviato T, Codrich D, Barbi E . Adenomyomatosis of the gallbladder as a cause of recurrent abdominal pain. J Pediatr 2018; 202: 328–28. doi: 10.1016/j.jpeds.2018.05.020 29903530

[64] Parolini F, Indolfi G, Magne MG, Salemme M, Cheli M, Boroni G, et al . Adenomyomatosis of the gallbladder in childhood: A systematic review of the literature and an additional case report. World J Clin Pediatr 2016; 5: 223–27. doi: 10.5409/wjcp.v5.i2.223 27170933 PMC4857236

[65] Nishimura A, Shirai Y, Hatakeyama K . Segmental adenomyomatosis of the gallbladder predisposes to cholecystolithiasis. J Hepatobiliary Pancreat Surg 2004; 11: 342–47. doi: 10.1007/s00534-004-0911-x 15549435

[66] Ootani T, Shirai Y, Tsukada K, Muto T . Relationship between gallbladder carcinoma and the segmental type of adenomyomatosis of the gallbladder. Cancer 1992; 69: 2647–52. doi: 10.1002/1097-0142(19920601)69:11<2647::aid-cncr2820691105>3.0.co;2-0 1571894

[67] Chao C, Hsiao HC, Wu CS, Wang KC . Computed tomographic finding in adenomyomatosis of the gallbladder. J Formos Med Assoc 1992; 91: 467–69. 1358320

[68] Yang HK, Lee JM, Yu MH, Lee SM, Park J, Han NY, et al . CT diagnosis of gallbladder adenomyomatosis: importance of enhancing mucosal epithelium, the “cotton ball sign.” Eur Radiol 2018; 28: 3573–82. doi: 10.1007/s00330-018-5412-4 29633001

[69] Tang S, Huang L, Wang Y, Wang Y . Contrast-enhanced ultrasonography diagnosis of fundal localized type of gallbladder adenomyomatosis. BMC Gastroenterol 2015; 15: 99. doi: 10.1186/s12876-015-0326-y 26239485 PMC4524444

[70] Joo I, Lee JY, Kim JH, Kim SJ, Kim MA, Han JK, et al . Differentiation of adenomyomatosis of the gallbladder from early-stage, wall-thickening-type gallbladder cancer using high-resolution ultrasound. Eur Radiol 2013; 23: 730–38. doi: 10.1007/s00330-012-2641-9 23247807

[71] Kim HJ, Park JH, Park DI, Cho YK, Sohn CI, Jeon WK, et al . Clinical usefulness of endoscopic ultrasonography in the differential diagnosis of gallbladder wall thickening. Dig Dis Sci 2012; 57: 508–15. doi: 10.1007/s10620-011-1870-0 21879282

[72] Maldjian PD, Ghesani N, Ahmed S, Liu Y . Adenomyomatosis of the gallbladder: another cause for a “hot” gallbladder on 18F-FDG PET. AJR Am J Roentgenol 2007; 189: W36-8. doi: 10.2214/AJR.05.1284 17579133

